# Retinal cytoarchitecture is preserved in an organotypic perfused human and porcine eye model

**DOI:** 10.1186/s40478-024-01892-y

**Published:** 2024-11-30

**Authors:** Darren Chan, Jenny Wanyu Zhang, Gah-Jone Won, Jeremy M. Sivak

**Affiliations:** 1 Donald K Johnson Eye Institute, Krembil Research Institute, University Health Network, Toronto, ON Canada; 2 Department of Ophthalmology and Vision Science, University of Toronto, Toronto, ON Canada; 3 Department of Laboratory Medicine and Pathobiology, University of Toronto, Toronto, ON Canada

**Keywords:** Ocular perfusion, Intravitreal drug delivery, Retinal cytoarchitecture

## Abstract

Pathobiology of the intact human retina has been challenging to study due to its relative inaccessibility and limited sample availability. Thus, there is a great need for new translational models that can maintain human retinal integrity and cytoarchitecture. The role of physiologic intraocular pressure (IOP) and fluid flow on retinal tissue has not been well studied. Here, we present an ex vivo organotypic model to assess the impact of physiological intraocular perfusion on retinal cytoarchitecture and cell survival. We demonstrate that retinal cytoarchitecture is remarkably well preserved following re-establishment of physiological IOP and aqueous humor dynamics for up to 24 h in ex vivo whole globe porcine and human eyes, comparable to freshly preserved control eyes. Accordingly, cell death was minimized in the perfused retinas, which also displayed normal markers of cellular metabolism and astrogliosis. These results are in marked contrast to contralateral control eyes without active perfusion, which displayed excessive cell death and disrupted cytoarchitecture at the same time point. These experiments demonstrate the critical impact that physiological pressure and fluid flow have on retinal tissue, and introduce a new pre-clinical model to study human and porcine retinal health and degeneration in a relevant biomechanical setting.

**Supplementary Information:**

The online version contains supplementary material available at 10.1186/s40478-024-01892-y.

## Introduction

Retinal degenerations account for the leading causes of irreversible blindness, worldwide [1–3] Yet, translational research on health and degeneration of the adult human retina and related cellular pathobiology has been hampered by limited access to patient samples, and constraints on clinical procedures [4–6]. In comparison, common animal models differ from the human eye in certain key anatomical features, such as the presence of a macula and/or fovea [7–10], formation of a proper lamina cribrosa [11, 12], variable retinal vascular patterning [13–15], and dramatic changes in tissue thickness and size [10, 16–18]; all of which differ in various species. These physical and biological barriers to studying the intact human retina make it challenging to characterize this tissue in detail and assess effective therapeutic and drug delivery strategies [19].

A variety of experimental models have been developed to study the human retina in lieu of the in situ eye [20, 21]. In particular, ex-vivo organotypic culture systems involve incubating ocular tissues in controlled conditions [22–24], thereby preserving the natural cytoarchitecture and cellular interactions of the retina. Retinal explant cultures, a common and clinically-relevant approach, have been widely used to study molecular mechanisms, drug delivery techniques, and cell transplantation therapies [25–32]. However, retinal explants do not fully recapitulate the complex in-vivo architecture of the posterior segment. Posterior cannulations of the retinal arteries in whole animal eyes address these issues [19, 33–39], but are impossible to achieve in enucleated human eyes due to irreversible coagulation. Retinal organoids generated from human embryonic or induced pluripotent stem cells have emerged as novel culture systems that retain the 3D organization of the retina and are amenable to genetic and pharmacologic manipulation [40–45]. Yet, key cytoarchitectural and biomechanical features are still compromised using this approach compared to the intact human retina [20, 42, 44–46]. Therefore, there remains an urgent need for new ex vivo models that can maintain human retinal integrity and cytoarchitecture.

In situ, the retina is subjected to a complex combination of biomechanical forces such those imposed by fluid convection and pressure of the vitreous body [47], which have unclear impacts on retinal tissue integrity and cell survival [48]. The outflow of aqueous humor in the anterior segment ultimately impacts these biomechanical properties of the posterior segment, as flow in the anterior chamber drives fluid convection in the vitreous body and maintains physiological intraocular pressure (IOP) [49–54]. In addition, disrupted aqueous flow can increases IOP and is a major risk factor for development of glaucoma [1, 55, 56]. To study these relationships in the context of vitreous drug particle movements we recently developed an ex vivo ocular perfusion technique [57], based on methods previously developed to re-establish anterior fluid dynamics with a synthetic aqueous substitute and preserve outflow tissue viability and pharmacology in animal models and human eyes [24, 57–64]. Our approach carefully recapitulates in vivo fluid dynamics and pressure in the posterior segment by re-establishing physiologic anterior fluid outflow [57]. We previously applied this system to favourably compare the in vivo and ex vivo distribution of drug particles and tracer beads in the posterior segment of porcine, non-human primate, and human eyes following intravitreal injection. During this work we noted that the retina appeared remarkably intact after several hours of perfusion, with no additional supplements or optimization. Yet, the impact of ocular fluid dynamics and pressure on retinal tissue integrity have not been well-studied. Therefore, we wondered if this system could be adapted to study the impact of pressure and vitreous flow on human retinal cytoarchitecture ex-vivo.

Here, we present a series of experiments to study and optimize the impact of organotypic ocular perfusion on retinal cytoarchitecture in porcine and human eyes. First, physiological IOP and outflow were re-established to restore ocular globe integrity and homeostasis. Then, assessments were made of retinal cytoarchitecture and markers of cell death, survival and metabolism. These experiments test the critical impact that physiological fluid flow and pressure have on retinal tissue, and introduce a model to study the human retina in a relevant cytoarchitectural and biomechanical setting.

## Materials and methods

### Tissue preparation

All animal experiments were performed in accordance with the ARVO Statement for the Use of Animals in Ophthalmic and Vision Research, and according to approved UHN Animal Use Protocols. Fresh porcine eyes were obtained from the University Health Network animal resource center within 6 h (h) of enucleation. All experiments using human tissue were performed according to protocols approved by the UHN Research Ethics Board and adhered to the Declaration of Helsinki. Donated human eyes were obtained through the Eye Bank of Canada (Ontario Division), and were intact healthy globes from donors aged from 22 to 67 years. Eyes were enucleated within 12 h and the tissue was received within 24 h of the time of death. Eyes with history of ocular tumors, vascular disease, or glaucoma were excluded. Human eyes were prepared, perfused, and assessed similarly to porcine eyes.

### For all eye preparations

Immediately upon receiving the enucleated eyes, extraocular tissues were carefully removed and the eyes were wrapped in compression gauze around the global equator. Wrapped eyes were oriented, anterior-side up, in 50 mL beakers and then immersed to the level of the limbus in a bathing buffer of sterile Dulbecco’s Phosphate-Buffered Saline (DPBS) (Sigma-Aldrich) supplemented with 5.5 mM glucose, or one of several alternative bathing media as noted in the text, including: Dulbecco’s Modified Eagle Medium (DMEM) with Low Glucose (Sigma), DMEM/Nutrient Mixture F12 (Multicell), and Neurobasal A Medium (NBA) with supplements (Gibco), (Supplementary Table 1). Beakers containing the eyes were then placed into a heated water bath prior to cannulation. Eyes were cannulated by puncturing a 25G needle (BD Vacutainer^®^) through the cornea, 2 mm anterior to the limbus. The needle was then guided to the posterior chamber by threading carefully through the gap between the iris and the lens. A second needle connected to synthetic aqueous humor (AH_syn_) reservoir was cannulated at the anterior chamber to maintain the IOP temporarily when the perfusion system was under adjustment.

### Ocular perfusion system

Anterior ocular perfusion was established as detailed in our previous publication [57]. Briefly, a two-channel system was used that included a motorized pulse-free syringe pump (PHD 2000, Harvard Apparatus) and dual pressure sensors, so that two eyes could be assessed simultaneously. The pump drove two 10 mL syringes loaded with infusion buffer of degassed DPBS supplemented with 5mM glucose as a simple synthetic aqueous humor equivalent (AH_syn_) into each eye at constant physiological rate of 2.4 µL/min for the indicated time. AH_syn_ was also sonicated for 30’ prior to use to minimize the emergence of air bubbles in tubing during perfusion. To determine eyes acceptable for study, we first established a stable perfusion for at least 30 min, and set an inclusion range for physiological IOPs between 12 and 18 mmHg. The perfusate was passed through a 0.2 μm syringe filter installed between each syringe and an infusion line of PE160 polyethylene tubing (Intramedic^®^, Becton Dickinson). Each infusion line was split by a three-way valve (Hamilton^®^) to incorporate a calibrated pressure sensor/transducer (142PC01G, Honeywell) to monitor pressure at the point of cannulation. Transducer output signals in voltage were recorded with a portable multi-channel data logger (OM-DAQPRO-5300, Omega). Note, the raw IOP readouts were absent the contribution of in-vivo episcleral venous pressure EVP [65]. Therefore, an additional calibration step accounted for EVP in the processed dataset. The water bath temperature of 38 °C was established previously to accurately reproduce the critical in vivo anterior-posterior temperature differential that contributes to the anterograde convection current at an average room temperature of 20 °C [57, 66]. As a proof of principle some eyes were subjected to non-invasive imaging using spectral-domain OCT; Heidelberg Eye Explorer 1.10.4.0 (Heidelberg Engineering GmbH, Heidelberg, Germany). This device combines confocal scanning laser ophthalmoscopy and conventional OCT technology. Immediately following perfusion, study eyes were gently wrapped in moist gauze and oriented in an imaging support so that the central retina and optic nerve head could be scanned.

### Confocal microscopy

Following perfusions, eyes were slit near the limbus and immersion fixed overnight in 4% (v/v) paraformaldehyde (PFA), then hemidissected through the equator, cryoprotected with 30% (w/v) sucrose solution and embedded in Optimal Cutting Temperature (OCT) compound (Sakura) for cryosectioning. The posterior segments were isolated and sectioned at 18 μm thickness. Retinal cell types were assessed with primary antibodies as previously described [67, 68], incubating with primary antibodies overnight at 4 °C, and secondary antibodies for 1 h at room temperature. Primary antibodies used were raised against RNA-binding protein with multiple splicing (RBPMS; Phosphosolutions), calbindin (Abcam), AP-2α (Developmental Studies Hybridoma Bank), CHX10 (Exalpha), β3-tubulin (Abcam), R/G-opsin (Abcam), glutamine synthetase (GS; Abcam). In addition, 4’,6-Diamidino-2-Phenylindole Dihydrochloride (DAPI; ThermoFisher) was used for visualization of cell nuclei (antibody sources and dilutions are listed in Supplementary Table 2). Changes to retinal morphology and marker signals were assessed qualitatively, and density of RGCs, Amacrine cells, and Bipolar Cells from central and peripheral retinal regions were counted and compared between groups. Furthermore, TdT-mediated dUTP Nick-End Labeling (TUNEL) assays were carried out on retinal sections according to the manufacturer’s directions to detect evidence of apoptosis (DeadEnd Colorimetric TUNEL System, Promega). TUNEL positive cells in each retinal layer were compared between 6 pairs of perfused vs. non-perfused eyes. For quantifications each eye had counts averaged from 4 peripheral sections of 200 μm (taken from 1 mm adjacent to the pars plana) and 4 central sections (taken from 1 mm adjacent to the optic nerve head) for comparison. Imaging was performed on a Nikon Ti2 Confocal Microscope. All images were acquired at 20X magnification and a resolution of 1024 × 1024 pixels, with the exception of Fig. 3E-F and Supplemental Fig. 2 at 10X, as indicated by corresponding scale bars.

**Fig. 1:**
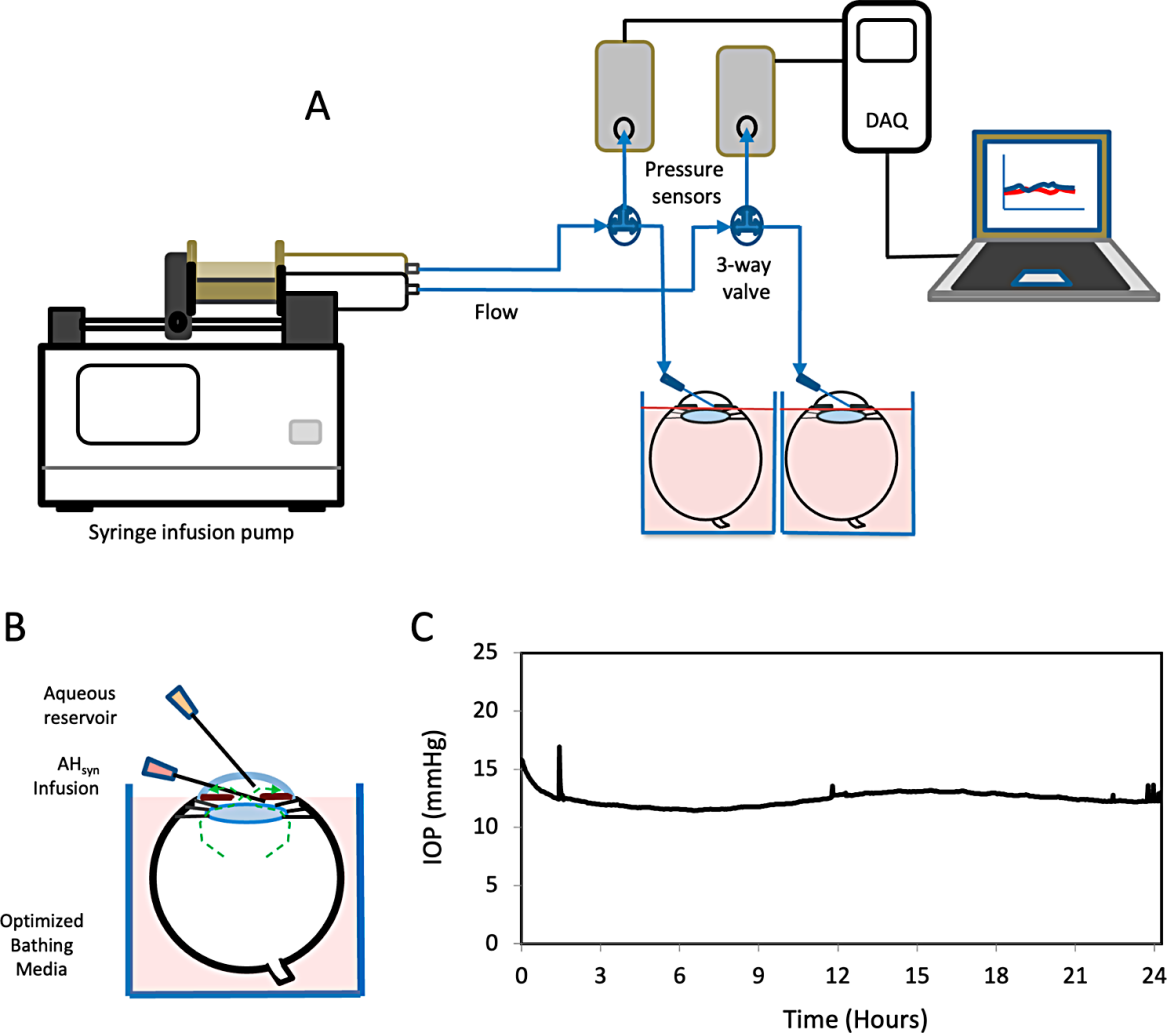
System design and establishment of stable physiological IOP. **A**) A cartoon schematic of the system shows a syringe pump infusing dual posterior chamber cannulations of eyes maintained in a bathing medium and maintained at a constant temperature in a water bath. The infusion lines are split by three-way valves to incorporate transducers to monitor pressure at the point of cannulation. The transducers are connected to a data logger for storage and readout. **B**) A cartoon cross section of an eye mounted in optimized bathing medium to the limbus, leaving the cornea exposed. The posterior chamber cannulation for AH_syn_ infusion, and aqueous sampling port are indicated. Dotted-line arrows indicate the resulting anterior convection flow (green). **C**) Representative physiological pressure recording over a 24 h perfusion

### Statistical analyses

Throughout, ‘n’ refers to individual eyes, as noted for each figure. The total number of porcine and human samples studied are specified in Table 1. All data were analyzed using a student’s T-test or ANOVA with Bonferonni post-hoc multiple comparisons test using Graphpad Prism 9 software.

**Table 1:** Eyes and species used in perfusion experiments

Figure	Total # of eyes used	Species	Total
2	18	Pig	33
3			
S3	15		
4	6	Human	6

## Results

### Retinal cytoarchitecture is preserved in anterior perfused porcine eyes

Based on our previous observations that retinal markers and architecture were intact after 4 h in anterior-perfused eyes [57], we wondered whether retinal tissue can be sustained over a more extended period ex vivo by using this approach in surrogate porcine eyes, and ultimately in donated human eyes.

Physiologic intraocular flow and pressure were re-established as previously described [57], and in the detailed methods. Briefly, physiological fluid convection was restored in enucleated eyes by a syringe pump infusing synthetic aqueous humor (AH_syn_) into the posterior chamber. An in-line pressure transducer connected to a data logger enabled continuous pressure monitoring at the site of cannulation (Fig. 1A). The eyes were immersed in a beaker containing a simple bathing media of DPBS with 5.5 mM glucose, raised to the level of the limbus and maintained at a constant temperature in a water bath. This arrangement leaves the cornea exposed to atmospheric temperatures to create a physiological temperature differential that promotes an important intraocular convection current [66] (Fig. 1B). An additional needle connected to a reservoir of AHsyn was inserted into the anterior chamber as an outlet for gas formed during perfusion (Fig. 1B). The infusion rate of AHsyn was maintained at a physiological flow rate of 2.4 µl/min, corresponding to average aqueous flow measurements. In healthy porcine globes [69, 70]. This infusion generally resulted in stable physiological EVP-corrected IOPs over 24 h of perfusion [58, 71–73] (Fig. 1C).

**Fig. 2:**
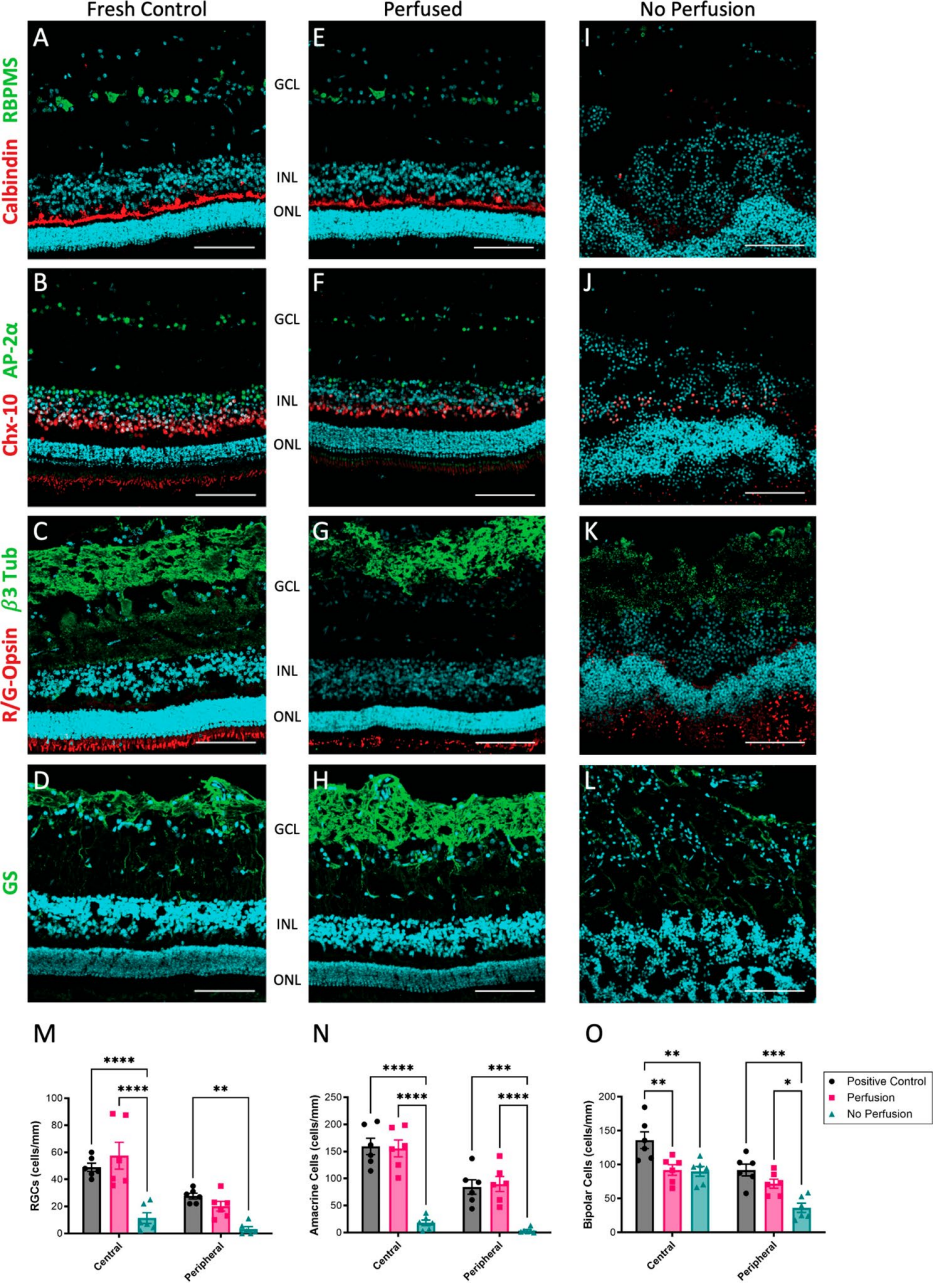
Retinal cytoarchitecture and cell survival are preserved in perfused eyes. Retinas from fresh porcine eyes (Control), 24 h anterior perfused eyes (Perfused), and 24 h no-perfusion control (No Perfusion) were sectioned and stained with antibodies directed to a variety of retinal cell markers, which were assessed for cytoarchitectural features. **A**,**E**,**I**) Representative images of RBPMS and Calbindin, **B**,**F**,**J**) Chx-10 and AP-2α, **C**,**G**,**K**) R/G-Opsin and β3-Tub, and **D**,**H**,**L**) GS. The corresponding markers and cytoarchitecture were preserved in retinas following 24 h of anterior perfusion compared to severely disrupted staining in eyes maintained under the same conditions, but without active perfusion (No-Perfusion). (Scale bars = 50 μm, GCL; ganglion cell layer, INL; inner nuclear layer, ONL; outer nuclear layer). **M**) Counts of RGCs (RBPMS staining) in both central and peripheral retina are not significantly different in perfused eyes compared to fresh controls, in contrast to significantly reduced numbers in non-perfused eyes (*n* = 6). **N**) Amacrine cells (AP-2α staining) and (**O**) bipolar cells (Chx-10 staining) similarly show significant preservation in perfused eyes compared to non-perfused (*n* = 6). In the central retina there was a significant loss of bipolar cells in perfused compared to fresh control eyes. (*p < 0.05, **p < 0.01, ***p < 0.005), bars are S.E)

Next we began a series of experiments to investigate the effect of physiological anterior perfusion on retinal cytoarchitecture. As a proof of principle that the perfused eyes remain clear for imaging, perfused porcine eyes were mounted for fundus imaging and ocular coherence tomography (OCT) through the central retina and optic nerve head (Supplementary Fig. 1). For further analyses retinal sections were prepared from porcine eyes and probed for specific immunofluorescent markers. These initial experiments were performed in fresh porcine eyes, which serve as a close human anatomical surrogate in many aspects [71, 74–76]. The contralateral eye was included as a negative control by assembling the apparatus and cannulating without activation of the perfusion pump. A positive control of freshly fixed eyes was also included for comparison of expected tissue architecture. Remarkably, after 24 h of perfusion, confocal micrographs revealed that perfused retinas maintained a relatively normal cytoarchitecture comparable to fresh frozen positive control tissue, with DAPI staining outlining a clear ganglion cell layer (GCL), inner nuclear layer, (INL), and outer nuclear layer (ONL) (Fig. 2A-H). In comparison, negative control (non-perfused) eyes exhibited dramatic tissue disruption, with loss of each layer’s distinction and integrity (Fig. 2I-L). As additional analyses, the survival of individual cell types were assessed using specific antibodies raised to markers for retinal ganglion cells (RGCs; RBPMS), horizontal cells (calbindin), Müller glia (glutamine synthetase; GS), amacrine cells (AP2α), bipolar cells (chx-10), neuronal filaments (β3-tubulin), and red/green cones (R/G-opsin). Confocal images were generated showing that each of these markers displayed representative cellular staining patterns and intensity compared to the fresh positive control samples (Fig. 2A-H). In comparison, for non-perfused eyes most markers were absent, with only bipolar cells and R/G-cones still present, though with a clearly disrupted signal (Fig. 2I-L, lower magnification images are presented in Supplementary Fig. 2). Such dramatic results were surprising, as only the anterior perfusion of AH_syn_ buffer was provided to differentiate the sample groups.

**Fig. 3:**
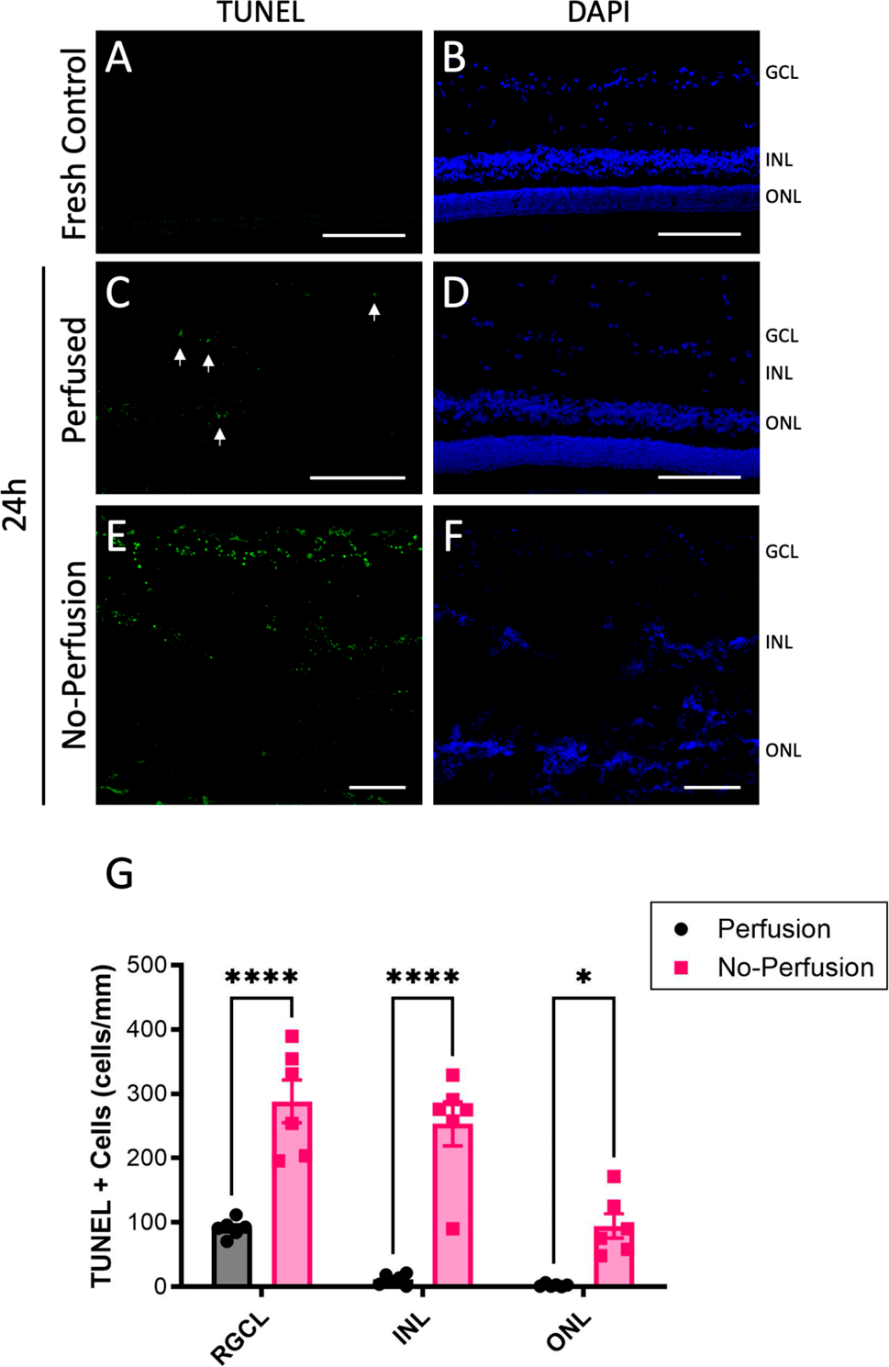
Retinal cell death is reduced by physiological ocular perfusion. **A**-**F**) Representative TUNEL (green) and DAPI (blue) and staining of central retinal sections after 24 h perfusion. There was no signal in fresh control retinas, some staining in perfused retinas after 24 h (arrows), but much more positive staining appeared in no-perfusion retinas (scale bars = 50 μm. Note; lower magnification was necessary to accommodate the highly disrupted no-perfusion images, as indicated by the scale bars). **E**) Quantification of the TUNEL signal from multiple eyes shows significantly less cell death in perfused eyes in each retinal layer when compared to no-perfusion eyes (*n* = 6). (*p < 0.05, **p < 0.01, ***p < 0.005, bars are S.E)

For quantitative analyses the relative counts of several key retinal cell types were compared from these images. Due to typical variations in cell density across the retina, cells were counted in central and peripheral regions for each marker analyzed in control, perfused, and non-perfused eyes. For RGCs, no differences were observed when comparing positive control and perfused eyes in the central (*p* = 1.000), or peripheral (*p* = 0.578) retina. However, retinal sections from non-perfused eyes showed dramatically fewer RGCs in the central and peripheral regions when compared to positive control (central: *p* < 0.001, peripheral: *p* = 0.003), and compared to perfused eyes (central: *p* = 0.001, peripheral: *p* = 0.065) (Fig. 2M). Similarly, for amacrine cells, no differences were observed when comparing positive control and perfused eyes in the central (*p* = 1.000) and peripheral (*p* = 0.578) retina. Yet, retinas from non-perfused eyes showed significantly fewer cells in both central and peripheral regions when compared to positive control (central: *p* < 0.001, peripheral: *p* = 0.003), and compared to perfused eyes (central: *p* = 0.001, peripheral: *p* = 0.003) (Fig. 2N). Finally, bipolar cells showed the same trend, with preserved cell numbers in perfused peripheral eyes compared to positive controls (*p* = 0.208), although a significant loss was detected in the central retina compared to positive controls (*p* = 0.002). Furthermore, non-perfused eyes exhibited significant loss of bipolar cells in the peripheral retina compared to positive control (*p* < 0.001) and perfused eyes (*p* < 0.002) (Fig. 2O).

### Cell death is reduced in anterior perfused porcine eyes

As an additional assessment of retinal damage, perfused and non-perfused retinal sections were probed by fluorescent terminal deoxynucleotidyl transferase dUTP nick-end labeling (TUNEL) assay, as a measure of active cell death. Following 24 h of perfusion, porcine retinas displayed a sparse TUNEL signal, particularly in the GCL and INL (Fig. 3B-C), compared to fresh control retinas, which displayed no TUNEL signal (Fig. 3A-B). However, in comparison, non-perfused control retinas displayed a massive TUNEL signal suggesting substantial apoptotic cell death in progress (Fig. 3E-F). Corresponding quantification counted TUNEL positive cells in each central retinal layer across multiple eyes. Significantly increased apoptosis was observed in non-perfused eyes compared to perfused eyes in each retinal layer (*p* < 0.005) (Fig. 3G). These data, together with cell staining data, indicate that the re-establishment of intraocular convection and physiological pressure preserves retinal cytoarchitecture and the cell death in porcine eyes.

### Optimized bathing media improves retinal cell survival over longer-term perfusions

So far, our studies only used a simple bathing media composed of DPBS supplemented with glucose. To better preserve retinal health in perfused eyes, we assessed the neurosupportive effects of different bathing media in the perfusion system. Media supplements are predicted to permeate the sclera [77, 78] and provide nourishment to the retina, as cannulations of human retinal vasculature are ultimately not possible due to irreversible coagulation. Because the study eyes have unavoidably undergone optic nerve transection during enucleation, RGC death is an unavoidable eventual result [79–82]. Therefore, we assessed the impact of several different media supplements at a later perfusion timepoint to minimize damage, specifically the loss of RGCs.

As before, porcine eyes were immersed to the level of the limbus in bathing medium. We compared four bathing media formulations: DPBS supplemented with 5.5mM glucose (composition of AH_syn_), Dulbecco’s Modified Eagle Medium (DMEM) with Low Glucose, DMEM/Nutrient Mixture F12 (optimized for astrocyte cultures), and Neurobasal A Medium (NBA) with supplements (optimized for RGC cultures, Supplemental Table 1). As the RGCs were well-preserved at 24 h (Figs. 2 and 3), to accentuate any potential media effects we extended the length of perfusions to 48 h. Compared to fresh porcine eyes, eyes bathed in DPBS or DMEM with Low Glucose at 48 h displayed a noticeable drop in retinal cytoarchitectural integrity, and a trend towards loss of RGC density (Supplementary Fig. 3A-C). Corresponding quantification of RBPMS staining showed a trend toward loss of RGC numbers (Supplementary Fig. 3F). In contrast, eyes bathed in F12 or NBA media demonstrated better preservation of cytoarchitecture. Notably, eyes bathed in NBA showed RGC counts comparable to those of the fresh control. (Supplementary Fig. 3D-F). Interestingly, although RBPMS intensity has not generally been assessed as a marker for RGC health, we noted that eyes bathed in NBA also had the brightest RBPMS signal in the GCL compared to other perfusion conditions. Therefore, retinal tissue survival appears to be enhanced by including an supportive bathing media.

### Retinal cytoarchitecture and cellular metabolism are preserved in organotypic perfused human eyes

To determine whether retinal cytoarchitecture might also be observed in organotypic human eyes, we performed a series of proof-of-principle perfusion experiments with freshly donated samples obtained from the Eyebank of Canada (Ontario Division). Donated eyes were prepared and perfused as previously for 24 h using an optimized bathing media. Following perfusion, retinas were sectioned and probed for similar markers as those used on porcine eyes.

**Fig. 4:**
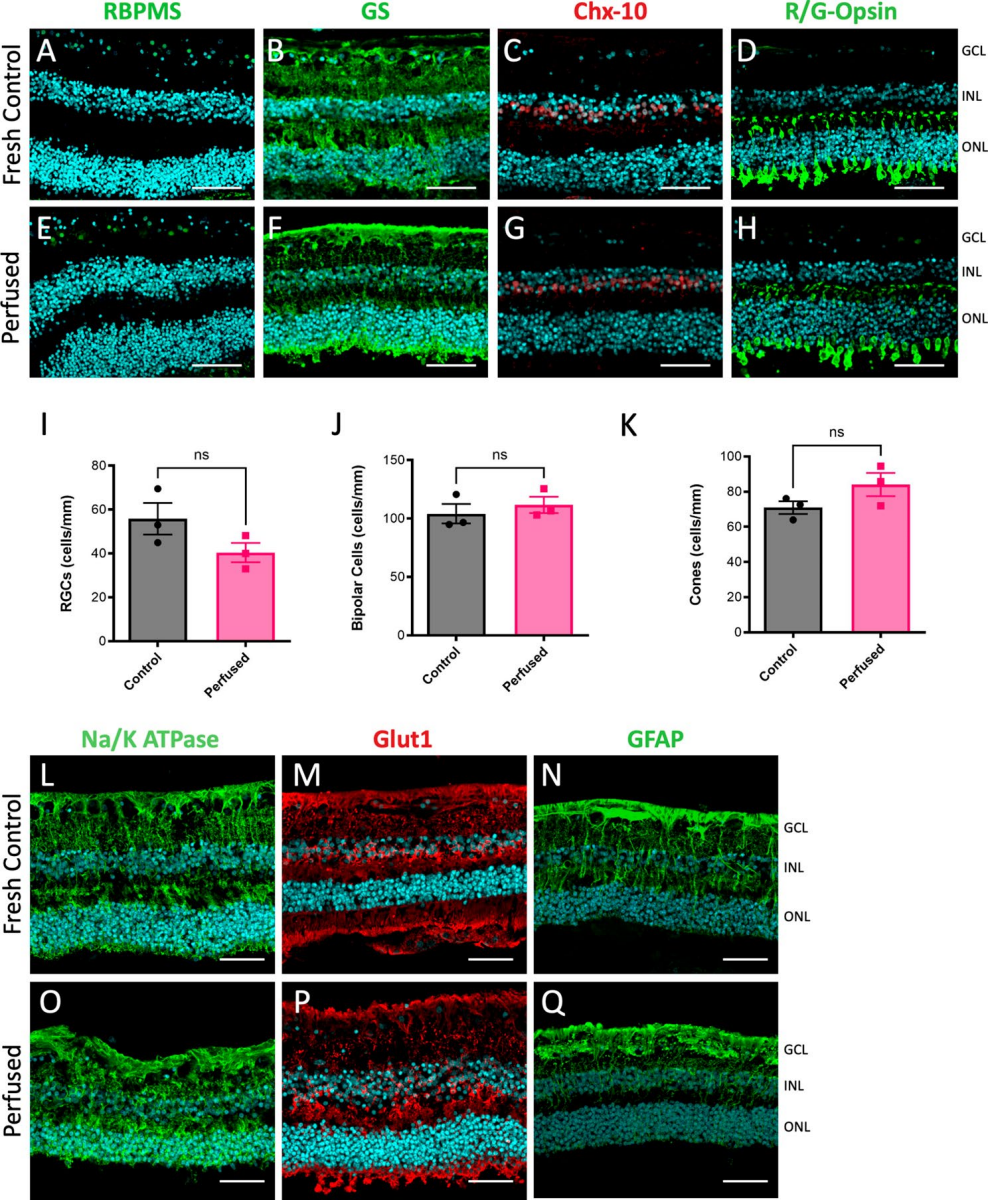
Retinal cytoarchitecture is preserved in perfused human eyes. **A**-**H**) A representative panel of human retina sections following 24 h perfusion (Perfused) in comparison to fresh frozen controls (Control). Preserved staining and morphology were observed for (**A**, **E**) RBPMS, (**B**, **F**) GS, (**C**, **G**) Chx-10, (**D**, **H**) R/G-Opsin (*n* = 3). Fresh control and perfused human retinas were counted for I) RGCs (RBPMS), J) bipolar cells (Chx-10), and K) cones (R/G-Opsin) with no significant differences observed (*n* = 3). (Scale bars indicate 50 μm). **L**-**Q**) Retinal tissue was probed for markers of active cellular metabolism (ATPase; **L**, **O**, Glut1; M, P) and glial reactivity (GFAP; **N**, **Q**). Similar antibody staining profiles were observed between fresh frozen (Control) and perfused human eyes (Perfused). (Scale bar = 50 μm)

Following perfusion, central retinal sections were stained positively for retinal ganglion cells (anti-RBPMS, Fig. 4A, E), Müller fibers (anti-GS, Fig. 4B, F), bipolar cells (anti-Chx-10, Fig. 4C, G), and R/G-cones (anti-R/G-opsin, Fig. 4D, H). Similar to the porcine retinas, localization of staining, and DAPI signal, maintained consistent morphology to sections from positive control fresh frozen retinas (Fig. 4A-D). Quantification of RGCs, bipolar cells, and cones showed no significant differences between fresh control and perfused human eyes (Fig. 4I-K). Additional staining for Na/K ATPase (Fig. 4L, O) and Glut-1 (Fig. 4M, P) suggested there were no differences in cellular metabolism markers between positive control and perfused eyes [83–85]. There was also no difference in GFAP staining for astrocyte reactivity (Fig. 4N, Q). Together, these results suggest that re-establishment of intraocular pressure and fluid dynamics also preserves retinal cytoarchitecture in organotypic perfused human eyes up to 24 h.

## Discussion

Here we have provided evidence that restoration of physiological anterior flow and IOP helps to maintain retinal tissue cytoarchitecture and cellular metabolism, initially in porcine and then donated human eyes. This approach is based on techniques to re-establish aqueous fluid dynamics in enucleated animal and human eyes through cannulation of the posterior chamber in order to assess outflow tissue responses of the anterior segment [24, 57–64]. Yet to our knowledge, the roles of physiological pressure and fluid convection on tissues of the posterior segment have not been well studied. In our previous work, we re-established physiological temperature, pressure, and flow parameters in ex-vivo whole porcine and human eyes to evaluate the distribution of intravitreally injected drug particles [57]. In this work, we noted that vitreous pH and overall retinal tissue integrity were remarkably maintained after short-term perfusion of the simple AHsyn formulation, without additional media or nutrient supplementation.

In the present detailed study, we assessed parameters of retinal cell type, cell survival or death, and cell metabolism markers in eyes that were perfused for 24 h, compared to contralateral negative controls without active perfusion, and positive fresh-fixed controls. Overall, in both human and porcine eyes, these analyses revealed perfused eye results that were comparable to freshly preserved control eyes, and were in marked contrast to retinas from eyes incubated in a parallel buffered system without perfusion, which became severely disrupted. This contrast occurred despite the presence of identical bathing media for the posterior globe in both groups, to permeate the sclera and provide support [77, 78]. Together, these results indicate that active ocular perfusion contributes to the striking protective effect. Yet, we note that the relationship between perfusion and pressure is interdependent and will be challenging to unravel experimentally. Active inflow is required to maintain pressure, and at present it is not possible to pressurize the eyes without perfusion. Disentangling the relative contributions of pressure and flow will require careful experimental design and modeling work.

Previously, other organotypic retinal perfusion approaches have been reported, such as tissue explants and cannulations of retinal vasculature [25–36]. However, these strategies either do not recapitulate defined intraocular fluid dynamics or tissue cytoarchitecture, or are not possible in human eyes due to irreversible occlusion of coagulated vessels. Taylor et al. recently demonstrated the importance of biomechanical strain on porcine retinal explant survival [48], and we have previously reported on the impact of biomechanical strain on retinal and optic nerve head glial cell function and survival [76, 86]. The retinal preservation we have observed here demonstrates the critical importance of physiological fluid dynamics and pressure on this tissue in situ, and are consistent with the recent work of Girkin et al. [87], who recorded structural and electrophysiological changes to increasing IOP in living human eyes of brain-dead donor patients that underwent anterior segment perfusion in situ.

We note that at present our model is limited in some parameters, such as the influences of episcleral venous pressure (EVP), retinal vascular flow and leakage, and retrolaminar cerebrospinal fluid (CSF) pressure. Future refinements may be able to introduce some of these variables. For example, since the eye is immersed in bathing media that imposes hydrostatic pressure, future work may model this parameter and compare it with physiological EVP. Diurnal fluctuation of IOP was also not modelled in our system, but can be included in subsequent experiments through programming the syringe pump to vary the rate of AH_syn_ infusion. Several additional parameters can also be isolated and optimized to improve culture viability and longevity. We note that extended perfusion to 48 h appeared to increase the incidence of retinal detachment and RGC cell loss. Although, it is unclear whether detachment occurred during perfusion, or during post-fixation processing. We showed that inclusion of additional nutrients and pro-survival factors in the bathing media can further prolong RGC survival from the optic nerve transection caused by enucleation [88, 89]. Additional points include temperature optimization, as lower temperatures have been reported to have beneficial impacts on retinal cell survival and storage [90–92]. Also additional optimizations of oxygenation, pH, and media may also be beneficial. In particular, the retina develops in a relatively hypoxic environment, and so it is unclear whether supplemental oxygenation will prove beneficial in this system [92–94]. In future work we aim to further optimize and refine the underlying relationships between anterior flow and retinal tissue responses to increase the effectiveness of the system in recapitulating the biomechanical environment of the living human eye.

Our findings also may have broader implications to help inform improved strategies for retinal explant and organoid cultures. Recent developments in 3D human retinal culture systems present opportunities to gain insights into human-specific retinal biology [40–46]. However, these systems generally lack the physiological fluid convection and imposed biomechanical strain of intact eyes. These culturing approaches may further benefit from the addition physiologically relevant fluid convection and biomechanical strain, in order to more closely resemble properties of intact human retina.

A successful organotypic whole-globe system that preserves retinal health would serve as a powerful preclinical model of the human retina. Given the substantial anatomical differences between common animal models and human eyes, there exists an unmet need for novel approaches that more closely mimic the physiological and biomechanical properties of the human retina. Our study demonstrates that through careful optimization of intraocular fluid dynamics, temperature, IOP and nutrients, the tissue integrity and cellular architecture of the human retina is preserved in enucleated human eyes during extended anterior perfusion. We are hopeful that with further optimization our approach might be improved to study retinal tissue responses in several preclinical and disease contexts, such as following intravitreal injections of drug formulations, or to assess the impact of altered fluid flow and IOP on retinal physiology, neuroinflammation and degeneration.

## Conclusions

We have optimized and characterized an ex vivo organotypic model in human, and surrogate porcine eyes, maintained through restoration of physiological fluid dynamics and IOP. With this approach retinal cytoarchitecture is remarkably well preserved, and cell death inhibited, for up to 24 h. These results are in marked contrast to contralateral control eyes without active perfusion, which display excessive cell death and disrupted cytoarchitecture. These experiments demonstrate the critical impact that physiological pressure and fluid flow have on retinal tissue, and introduce a new pre-clinical model to study human and porcine retinal health and degeneration in a relevant biomechanical setting.

## Electronic supplementary material

Below is the link to the electronic supplementary material.

Supplementary Material 1

Supplementary Material 2

Supplementary Material 3

Supplementary Material 4

Supplementary Material 5

## Data Availability

The main data supporting the findings of this study are available within the Article and its Supplemental Information. The raw data generated in this study are available from the corresponding author upon reasonable request.

## Abbreviations

AH_syn_: Synthetic aqueous humour
CSF: Cerebrospinal fluid
DAPI: 4’,6-diamidino-2-phenylindole dihydrochloride
DMEM: Dulbecco’s modified eagle medium
DPBS: Dulbecco’s phosphate buffered saline
EVP: Episcleral venous pressure
GCL: Ganglion cell layer
GS: Glutamine synthetase
INL: Inner nuclear layer
IOP: Intraocular pressure
NBA: Neurobasal A medium
OCT: Optimal cutting temperature compound
ONL: Outer nuclear layer
PFA: Paraformaldehyde
RBPMS: RNA-binding protein with multiple splicing
RGC: Retinal ganglion cell
TUNEL: Terminal deoxynucleotidyl transferase dUTP nick end labeling
