# Supplementary material for: Retinal cytoarchitecture is preserved in an organotypic perfused human and porcine eye model
Source: Acta Neuropathol Commun. 2024 Nov 30;12:186. doi: 10.1186/s40478-024-01892-y (PMC11607936; doi:10.1186/s40478-024-01892-y)
Supplement: Supplementary file 1 — Supplementary Material 1 [file 40478_2024_1892_MOESM1_ESM.docx]

| **Component** | **Product Code/Vendor** | **Concentration in Media** |
| --- | --- | --- |
| MACS NeuroBrew-21 (50X) | Miltenyi 130-093-566 | 1X |
| Glutamax (100X, 200mM) | Gibco 35050061 | 1mM |
| Human Recombinant BDNF (1mg/mL) | Gibco PHC7074 | 50 ng/mL |
| Rat Recombinant CNTF (1mg/mL) | Gibco PRC7015 | 50 ng/mL |
| Forskolin (6.09mM stock, 500uM working solution, 82.10 +917.9 uL H2O) | Bioshop FRS393.10 | 5uM |
| Penicillin, Streptomycin | Gibco 15140122) | 4% |
| FBS |  | 2% |
| HEPES | Bioshop HEP003.100 | 2% |

Supplementary Table 1. NBA media supplements.
