# Supplementary material for: Retinal cytoarchitecture is preserved in an organotypic perfused human and porcine eye model
Source: Acta Neuropathol Commun. 2024 Nov 30;12:186. doi: 10.1186/s40478-024-01892-y (PMC11607936; doi:10.1186/s40478-024-01892-y)
Supplement: Supplementary file 2 — Supplementary Material 2 [file 40478_2024_1892_MOESM2_ESM.docx]

| **Antibody** | **Catalog #** | **RRID** | **Dilution** |
| --- | --- | --- | --- |
| **Primary Antibodies** |  |  |  |
| Mouse Monoclonal anti Calbindin | ab82812 | AB_1658451 | 1:100 |
| Rabbit Polyclonal anti RBPMS | 1830-RBPMS | AB_2492225 | 1:200 |
| Sheep Polyclonal anti Chx10 | X1179P | AB_2889828 | 1:100 |
| Mouse Monoclonal anti AP-2 alpha | DSHB Cat# 3b5 | AB_528084 | 1:200 |
| Rabbit Polyclonal anti R/G Opsin | ab5405 | AB_177456 | 1:200 |
| Mouse monoclonal anti Beta-3-tubulin | ab78078 | AB_2256751 | 1:400 |
| Rabbit Polycloncal anti-Glutamine Synthetase | ab49873 | AB_880241 | 1:400 |
| Mouse Monoclonal anti Na+/K+ ATPase | sc-48345 | AB_626712 | 1:250 |
| Rabbit Polyclonal anti Glut1 | ab15309 | AB_301844 | 1:200 |
| Rabbit Polyclonal anti GFAP | ab7260 | AB_305808 | 1:400 |
|  |  |  |  |
| **Secondary Antibodies** |  |  |  |
| Donkey Anti-Rabbit Secondary Antibody, Alexa Fluor 488 | a21206 | AB_2535792 | 1:800 |
| Donkey anti-Rabbit Secondary Antibody, Alexa Fluor 568 | a10042 | AB_2534017 |  |
| Donkey Anti-Mouse Secondary Antibody, Alexa Fluor 488 | a21202 | AB_141607 |  |
| Donkey anti-Mouse Secondary Antibody, Alexa Fluor 568 | a10037 | AB_11180865 |  |
| Donkey anti-Sheep Secondary Antibody, Alexa Fluor 546 | a21098 | AB_2535752 |  |

Supplementary Table 2. Antibodies and dilutions used in immunofluorescent staining.
