# Supplementary figures and images for: Retinal cytoarchitecture is preserved in an organotypic perfused human and porcine eye model
Source: Acta Neuropathol Commun. 2024 Nov 30;12:186. doi: 10.1186/s40478-024-01892-y (PMC11607936; doi:10.1186/s40478-024-01892-y)

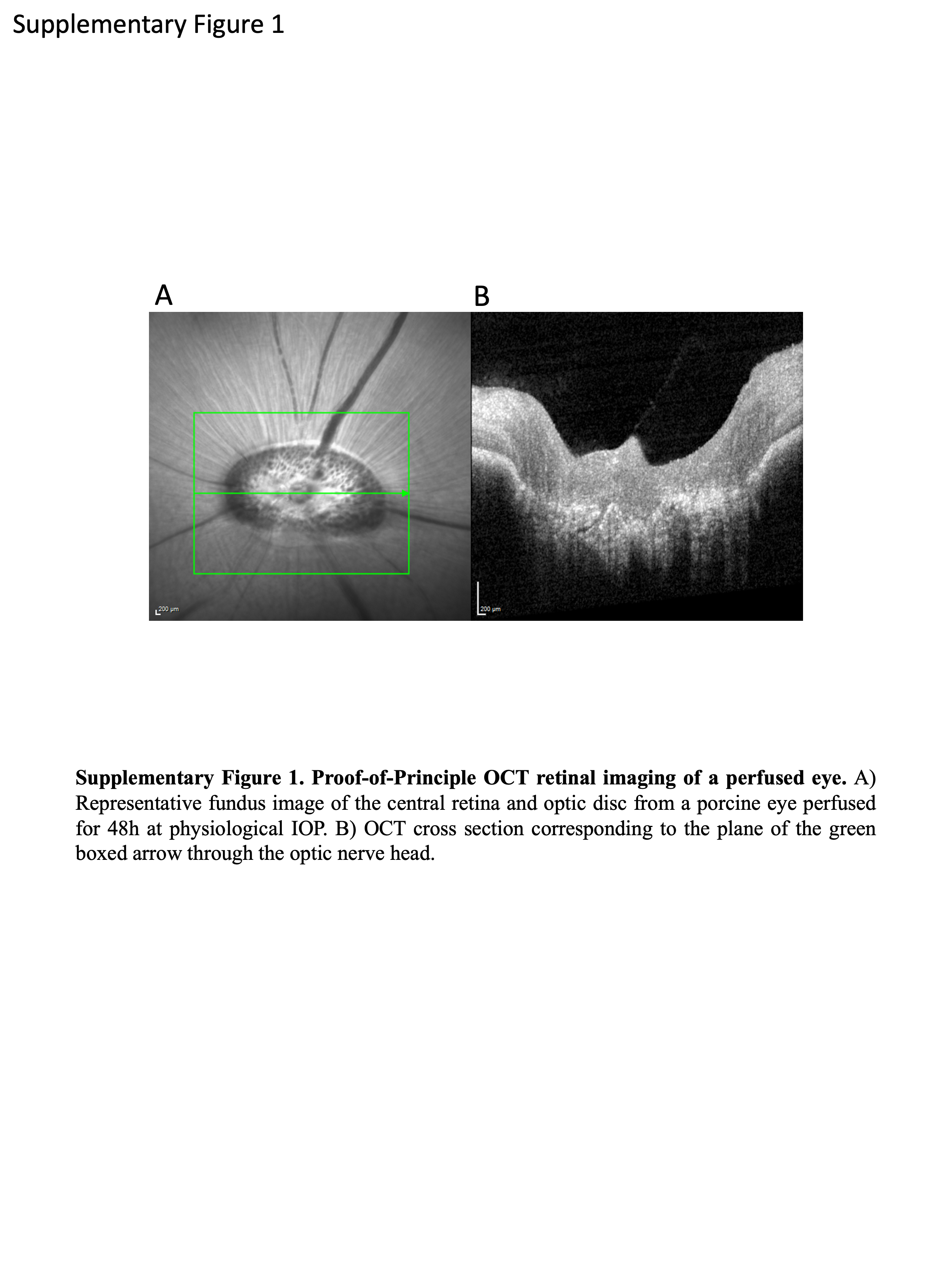

Supplement: Supplementary file 3 — Supplementary Material 3 [file 40478_2024_1892_MOESM3_ESM.tiff]

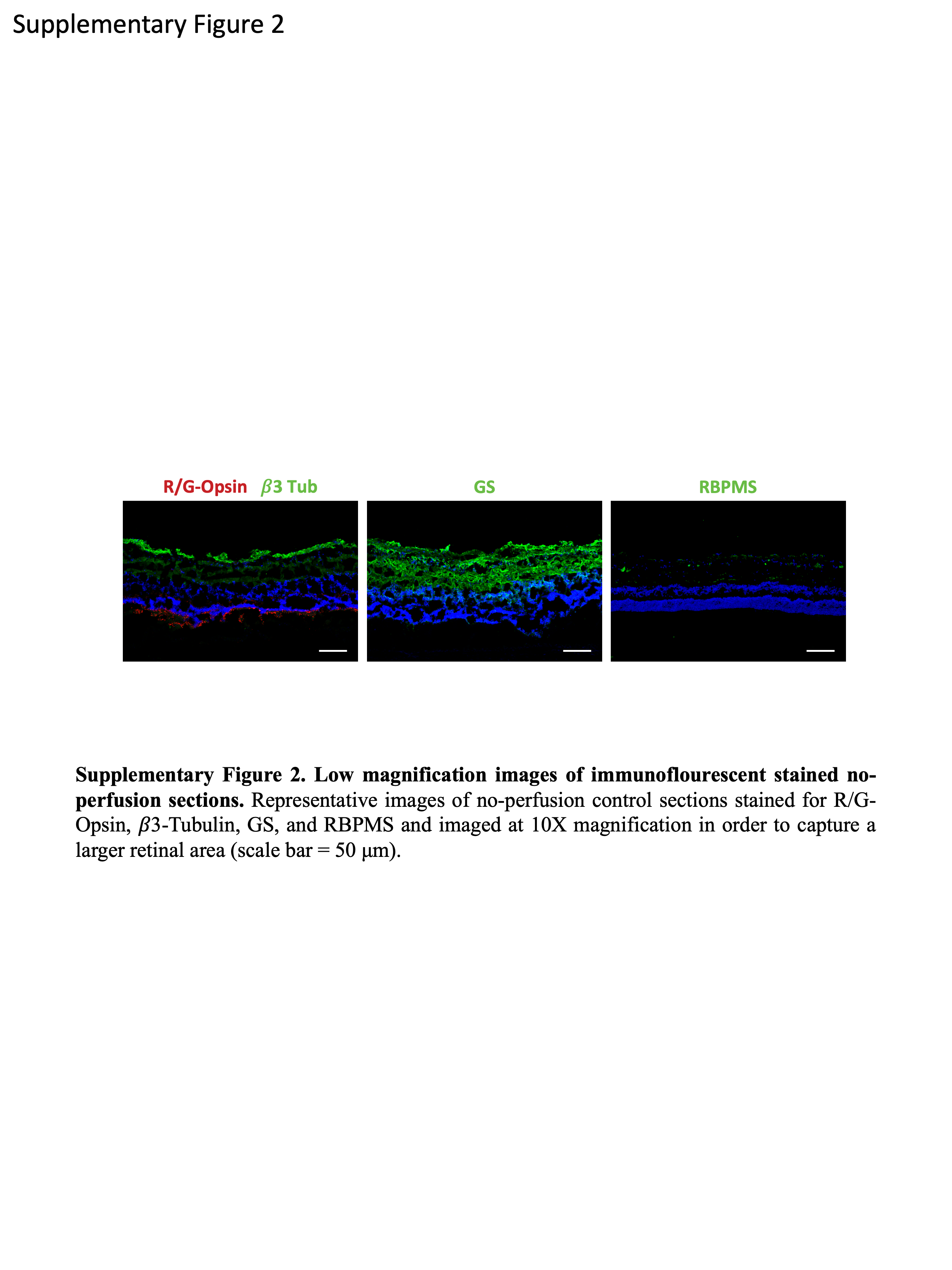

Supplement: Supplementary file 4 — Supplementary Material 4 [file 40478_2024_1892_MOESM4_ESM.tiff]

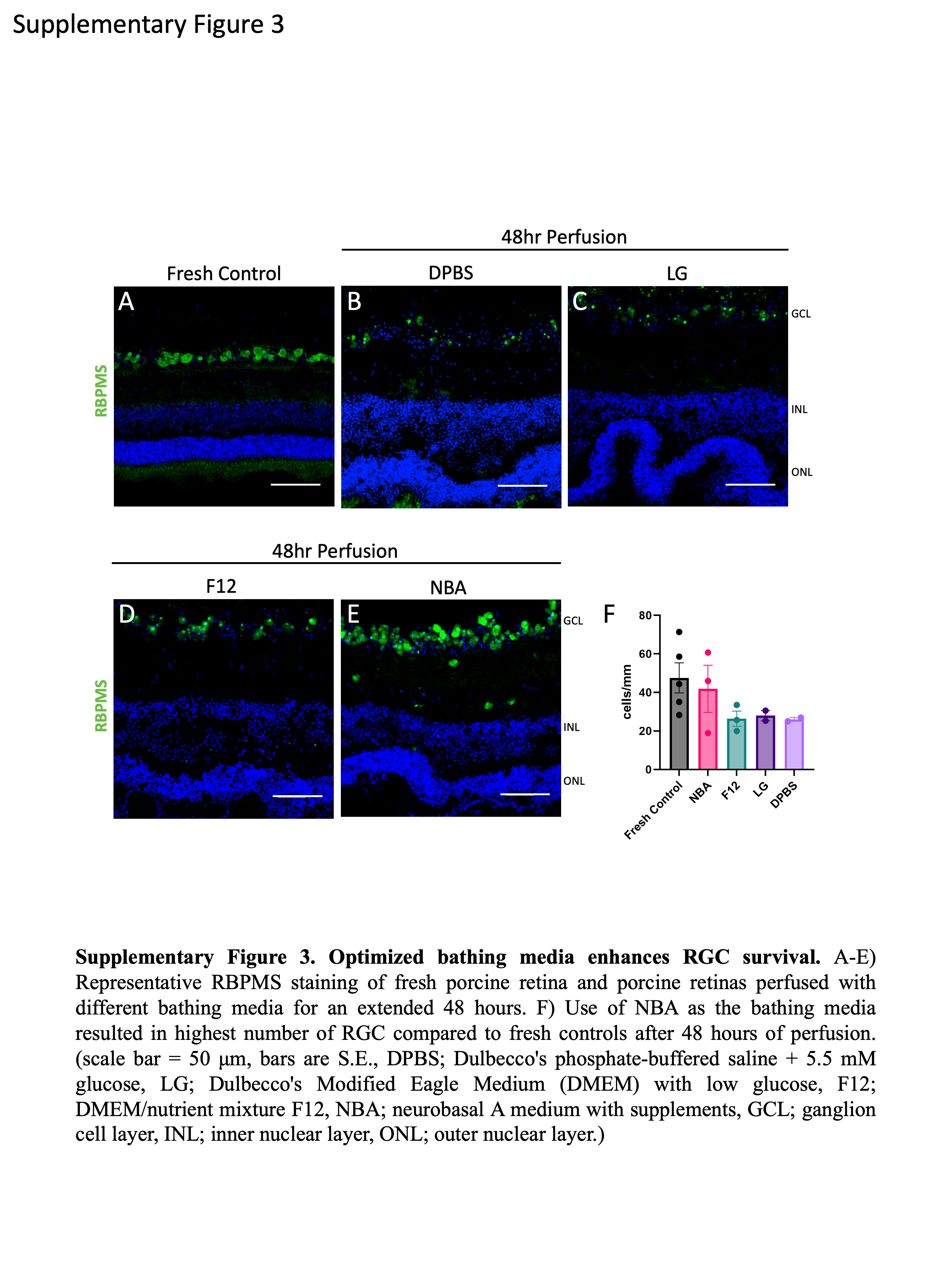

Supplement: Supplementary file 5 — Supplementary Material 5 [file 40478_2024_1892_MOESM5_ESM.tiff]
